# Development of an SPRi Immune Method for the Quantitative Detection of Osteopontin

**DOI:** 10.3390/s25123628

**Published:** 2025-06-09

**Authors:** Anna Sankiewicz, Beata Żelazowska-Rutkowska, Tomasz Guszcz, Ewa Gorodkiewicz

**Affiliations:** 1 Bioanalysis Laboratory, Faculty of Chemistry, University of Bialystok, Ciolkowskiego 1K, 15-245 Bialystok, Poland; ewka@uwb.edu.pl; 2 Department of Pediatric Laboratory Diagnostics, Medical University of Bialystok, Waszyngtona 17, 15-274 Bialystok, Poland; zelazowskab@wp.pl; 3 Department of Urology, Hospital of the Ministry of Interior and Administration in Bialystok, Fabryczna 27, 15-471 Bialystok, Poland; tomasz.guszcz@o2.pl

**Keywords:** ostepontin, surface plasmon resonance imaging (SPRi) biosensor

## Abstract

**Highlights:**

- SPRi biosensors for osteopontin determination have been developed.
- The bioreceptors are mouse or rabbit monoclonal anti-osteopontin antibodies attached to the gold surface of a chip via a cysteamine linker.
- The biosensors have sufficient sensitivity to determine osteopontin at levels characteristic of this protein in blood plasma without any signal enhancement.

**Abstract:**

Osteopontin (OPN) is a protein that plays many essential functions in the human body. It is present in most tissues and body fluids. OPN, among other things, participates in wound healing, the formation and remodeling of bone, immune response, inflammation, angiogenesis, and tumor formation. A new analytical method, based on SPRi (surface plasmon resonance imaging) biosensors, has been developed to determine osteopontin in biological fluids. OPN was captured from a solution by an immobilized antibody (mouse or rabbit), a bioreceptor in the SPRi sensor. A separate validation process was carried out for each antibody used. The LOD and LOQ values obtained for the biosensor with mouse antibody were 0.014 ng mL^−1^ and 0.043 ng mL^−1^, respectively, and those obtained for the biosensor with rabbit antibody were 0.018 ng mL^−1^ and 0.055 ng mL^−1^, respectively. The response ranges of both biosensors were in a similar range: 0.05–1.00 ng mL^−1^. OPN was determined in blood plasma to demonstrate the sensor potential, showing good agreement with the data obtained using an ELISA test and reported in the literature. The presented method is characterized by ease and speed of measurement, and the process does not require special preparation of samples.

## 1. Introduction

Studies of circulating biomarkers may provide additional helpful information in the diagnosis of various diseases, e.g., assessing the severity of the disease, monitoring the effectiveness of treatment, and predicting recurrence. For many years, there has been a search for substances specific to a given disease, with the concentration changing as the disease intensifies or subsides. The extracellular matrix (ECM) plays an important role in the development of many diseases. The ECM proteins play a key role in the regulation of numerous cellular functions. The ECM includes structural proteins, e.g., collagen and elastin, and specialized proteins, such as fibronectin, laminins, proteoglycans, and matricellular proteins. Matricellular proteins are nonstructural ECM proteins that perform regulatory functions through interactions with cell surface receptors, structural proteins, or soluble extracellular factors (growth factors, cytokines). Matricellular proteins include osteopontin (OPN), osteonectin, tenascins, and thrombospondins [1].

Osteopontin (OPN) is an acidic phosphorylated glycoprotein consisting of approximately 300 amino acid residues (314 amino acid residues in humans). It is a highly negatively charged protein. The mass of osteopontin is 33 kDa, but due to numerous post-translational modifications, its molecular mass can increase to approximately 45–75 kDa [2]. The human osteopontin gene consists of seven exons on the long arm of chromosome 4q21–25 [3]. OPN belongs to the SIBLING (Small Integrin-Binding LIgand N-linked Glycoprotein) family and is one of the primary mediators of extracellular matrix (ECM)–cell interactions [1,2].

**Figure 1:**
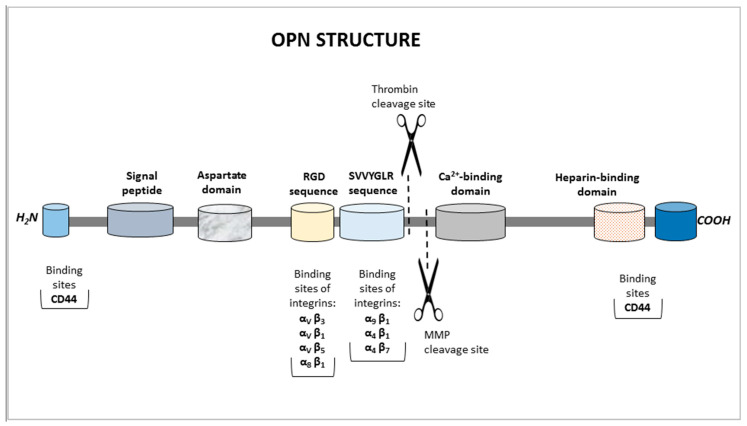
Schematic diagram of the structure of human osteopontin.

Osteopontin is a multi-domain protein. Its structure contains the NH2 and COOH-terminal regions, a signal peptide, an aspartate domain, an RGD sequence (Arg-Gly-Asp), an SVVYGLR sequence (Ser-Val-Val-Tyr-Gly-Leu-Arg), a thrombin cleavage site, a matrix metalloproteinase (MMP) cleavage site, and calcium- and heparin-binding domains (Figure 1) [3]. Each domain has different functions. The RGD motif interacts with cell surface αvβ3, αvβ1, αvβ5, and α5β1 integrins, while the SVVYGLR domain facilitates interactions with integrins α9β1 and α4β1. The aspartate domain binds with hydroxyapatite, and the domain located within the C-terminal fragment of the OPN molecule generated by thrombin can bind the hyaluronic acid receptor (CD44) [4].

Due to the fact that osteopontin interacts with many commonly expressed receptors on the cell surface, it is involved in numerous physiological and pathological processes. This protein is essential in the processes of activation, adhesion, migration, and survival in many cells. Among other things, it participates in the processes of wound healing, the formation and remodeling of bone, immune response, inflammation, angiogenesis, and tumor formation [5]. In particular, cancer development is associated with the increased secretion of OPN. Osteopontin induces various cell signaling pathways by binding to integrins or CD44 receptors. Through binding to αvβ3-integrin, the activation of NFκB (dependent on phosphoinositide (PI) 3-kinase/Akt) and the secretion of urokinase plasminogen activator (uPA) in cancer cells occur. The interaction of OPN with the CD44 receptor promotes cell survival and motility by activating the PLC-γ/PKC/PI 3-kinase pathways. In general, elevated levels of OPN contribute to tumor development by inhibiting apoptosis and activating various matrix-degrading proteases. This leads to increased tumor cell motility, tumor growth, and metastasis. Park et al. showed that advanced disease states of bladder cancer had higher circulating levels of osteopontin (stage T1 versus stage T2 and T2 versus T3) and were associated with a worse prognosis [6]. In many studies [4,5,6,7,8,9], OPN levels are associated with poor prognosis in cancers. It is considered a promising diagnostic and prognostic biomarker.

OPN is secreted into bodily fluids such as blood, urine, milk, semen, saliva, and cerebrospinal fluid [7,8,9]. Searching for an appropriate biomarker is essential in diagnosing many diseases. It is, therefore, important to develop simple methods for testing biological materials, especially those that are simple to collect, such as saliva, urine, or blood.

Currently, the most commonly used method is the enzyme-linked immunosorbent assay (ELISA). However, these tests are time-consuming and expensive, requiring a sizable sample volume, and the need for labeling is an additional disadvantage of immunoassays. The ELISA test involves immobilizing a target biomolecule (antigen) to a solid surface coating with a capture antibody that binds to the antigen. This antigen is complexed with a second specific antibody linked to a label or tag for detection. Binding is detected via a secondary reaction produced by the label and measured with readers. This technique is multi-step and requires the application of a chromogenic reaction using the appropriate substrate. In response, it produces a color whose intensity can be measured by a spectrophotometer or ELISA reader. The ELISA test permits a high degree of detection customization but comes with longer hands-on time requirements.

Alternatively, surface plasmon resonance (SPR) technology may be used. The phenomenon of surface plasmon resonance became interesting in the late 1970s. The potential of the phenomenon to study and observe processes occurring at the metal/dielectric interface due to the excitation of surface electrons was noticed. Currently, the SPR technique is most often used to study biomolecular interactions, opening up new possibilities for detecting biomarkers [10,11,12,13]. Combining the SPRi technique with a biosensor may allow the quantitative determination of a potential biomarker with high sensitivity and specificity [14,15]. SPRi experiments are simplified. Most often, SPRi assays involve the immobilization of a binding biomolecule (ligand) on the sensor surface, followed by injecting the analyte over the sensor surface. Preparing the biosensor does not require many steps, significantly reducing the response time in SPRi experiments. In addition, the possibility of regenerating the biosensor allows it to be used several times. SPRi tests offer economic advantages, as they save the cost of labeling reagents, and the small volumes of the reagents used reduce waste.

Several such applications can be found in the literature. Several biosensors for the determination of osteopontin have already been constructed. These include, for example, a lateral flow biosensor (LFB) [16], a paper-based aptasensor with colorimetric detection [17], an aptamer-based surface-enhanced Raman scattering and lateral flow assay (SERS–LFA) biosensor [18], and an electrochemical aptasensor for human osteopontin detection using a DNA aptamer selected by SELEX [19].

Our studies focus on the development of SPRi biosensors for the detection of osteopontin levels in body fluids. The proposed biosensor is based on the Kretschmann configuration for stimulating surface plasmons (SPs), which are electron density waves propagating at the metal and dielectric interface. To excite the surface plasmon waves (SPWs), the incident light should oscillate with the free electrons on the metal surface, causing them to resonate [20]. The phenomenon of surface plasmon resonance is sensitive to changes occurring on the metal surface. When subsequent layers are applied to the metal, the angle that causes the SPR effect shifts. These changes are proportional to the number of molecules adsorbed on the surface. This allows the detection and determination of the amount of a given substance. The Kretschmann configuration, which efficiently links evanescent waves to surface plasmons, significantly enhances the detection sensitivity and allows for the real-time observation of the arranged layers. This type of system allows for the simultaneous determination of leptin, fibronectin laminin-5, and type IV collagen in serum with high sensitivity and specificity [14,15].

The constructed biosensor is based on a specific antibody for osteopontin and the SPR method in the imaging version. In this version, measurements are carried out at a constant angle value, and the signals are recorded as an image [14,15]. The antibody immobilized on the metal surface by the linker (cysteamine) allows the detection of the analyte without cleaning and isolating the samples before measurement. There is no need to use labels, either. The sensor’s sensitivity, specificity, reproducibility, and reusability were evaluated. The method was also validated by measuring the OPN concentration in natural samples.

## 2. Materials and Methods

### 2.1. Materials and Reagents

Recombinant human osteopontin protein, monoclonal mouse (ab69498) and rabbit (ab214050) antibodies raised against human osteopontin, and the ELISA kit for osteopontin were supplied by Abcam (Cambridge, UK). Fibronectin, collagen type IV, albumin, cysteamine hydrochloride, N-ethyl-N′-(3-dimethylaminopropyl) carbodiimide (EDC), and N-hydroxysuccinimide (NHS) were from SIGMA-ALDRICH (Munich, Germany). The absolute ethanol, acetic acid, hydrochloric acid, sodium hydroxide, sodium chloride, sodium carbonate, and sodium acetate (POCh, Gliwice, Poland) were acquired from POCh (Gliwice, Poland). Phosphate-buffered saline (PBS) with pH = 7.4 from BIOMED (Lublin, Poland) was used to prepare solutions. Argon N 5.0 with an Ar content of 99,999% from AIR LIQUIDE Polska Sp. z o.o. (Krakow, Poland) was used. The chips covered with gold were acquired from Ssens (Enschede, The Netherlands). The biological material used in the research consisted of blood plasma samples from patients with bladder cancer and benign prostate hyperplasia (BPH). The samples were obtained from the Ministry of Interior and Administration Hospital in Bialystok (Poland). The blood was collected in EDTA tubes in the morning and was then centrifuged. The plasma was frozen at 800C. The study was approved by the local ethics committee of the Medical University of Bialystok (Poland) (APK.002.407.2024). All patients provided written informed consent for this study.

### 2.2. SPRi Measurement Methodology

The research used the SPRi spectrometer, which was constructed at the Bioanalysis Laboratory of the University of Bialystok in cooperation with the Bialystok University of Technology and AC S.A. The apparatus has been described in a previous publication [21].

The SPRi spectrometer consists of the following elements: a diode laser (λ = 635 nm) as a light source, a system of lenses that focus the incident radiation, and polarizers responsible for the p-polarization of the radiation. The next element is a holder in which an equilateral glass prism (BK-7glass) is placed, on which a drop of immersion oil with a refractive index consistent with the refractive index of the prism is placed. Next, the biosensor is placed on the prism. After passing through the optical elements, the radiation is reflected from the biosensor surface and goes to the detector (monochromatic CCD camera) and then to the computer. The signal is recorded as images, from which the final SPRi signal is calculated using ImageJ 1.51k software (NIH Image). All of the optical elements and the CCD camera were mounted on moveable arms, which allowed the angle of incidence of light on the biosensor to be set. The measurement is performed twice: first, a photo of the bioreceptor layer is taken, and then the layer is obtained after capturing the analyte. The signals are measured at the same angle as the incidence of light.

### 2.3. Sensor Biofunctionalization

To provide selectivity toward osteopontin, the gold surface of the glass chip was biofunctionalized based on the interaction of monoclonal mouse or rabbit antibodies with osteopontin. The gold chip was covered with a polymer mask to isolate gold active sites. A linker layer (cysteamine) was created on the gold. For this purpose, the gold chip was immersed in a 20 mM alcoholic cysteamine solution for 12 h at room temperature. After removing the chip from the solution, rinsing it with ethanol, and drying it, the next stage occurred, i.e., creating the osteopontin capture layer (antibody). To bind the antibody to cysteamine, the solution antibody was activated with a mixture of NHS (c = 250 mM), EDC (c = 250 mM), and carbonate buffer with pH = 8.5 in a volume ratio of 1:5:5:3. The mixture (3 μL) was applied to the active sites with a cysteamine linker layer. This solution was left for 5 min, and then the excess was collected to remove unbound biomolecules. The surface was rinsed 3 times with water and dried. In this way, an antibody layer was obtained, which was intended to capture OPN from the tested sample. Non-specific adsorption was eliminated using a 1 ng mL^−1^ bovine serum albumin (BSA) solution applied to one active site on each biosensor used.

### 2.4. Sensor Regeneration

After each measurement, the biosensor was cleaned as described [22]. The measurement chips were washed with a mixture of Triton X-100 (1%) containing NaOH (100 mM). Then, they were rinsed in distilled water and dried in a stream of argon. Due to alkaline hydrolysis, the deposited bioreceptor (antibodies) and the analyte layer were removed from the sensor surface. The chips prepared in this way were regenerated to the thiol layer and reused. The sensor with a thiol monolayer could be reused five times for the creation of a biosensor.

### 2.5. ELISA Measurements

The osteopontin concentration was determined using a human osteopontin ELISA kit according to the manufacturer’s instructions. This sandwich assay type has a concentration range of 109.38–7000 ng mL^−1^ and a sensitivity of 88 ng mL^−1^.

## 3. Results and Discussion

### 3.1. Formation of Layers on the Biosensor Surface

The first stage of the research was to confirm whether successive layers of the biosensor were formed on the surface of the gold plate. For this purpose, the changes in reflectance were investigated, which varied depending on the SPR angle after creating subsequent layers. A layer of cysteamine was created on the gold layer, and the first measurement was taken. Subsequent measurements were performed after applying an activated antibody (concentration of 30 ng mL^−1^) to the cysteamine layer and after the antibody interacted with osteopontin at a 1 ng mL^−1^ concentration. Details of the procedure are described in Section 2.3.

**Figure 2:**
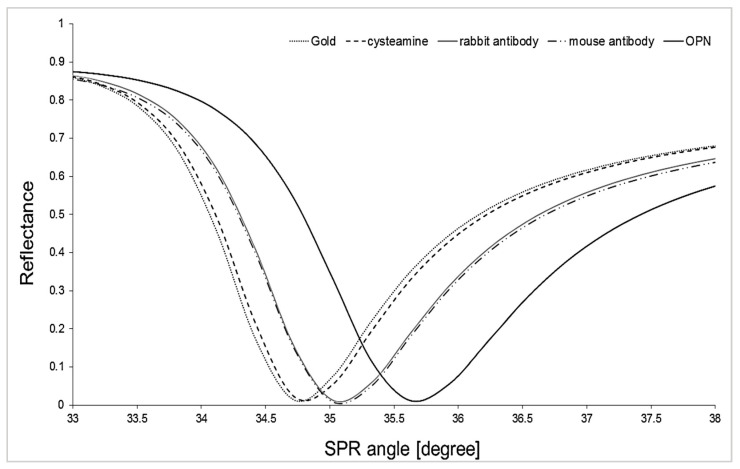
The course of model SPR curves. Data were collected at angles from 33 to 38 degrees for each distinguished layer on the biosensor surface, in 0.1-degree increments.

Then, SPR curves were fitted to the experimentally obtained data using WinSpall software (version 3.02, Res-Tec, Rosenheim, Germany). The results are presented in Figure 2. Every SPR curve shifts to higher SPR angle values than the curve obtained for the previous layer, indicating the presence of newly formed layers. Data acquisition was performed for each layer at 33- to 38-degree angles. The SPR curves obtained for mouse and rabbit antibodies overlap due to the very similar masses of these two antibodies.

### 3.2. Optimization of Experimental Parameters

#### 3.2.1. Selecting the Bioreceptor Concentration

**Figure 3:**
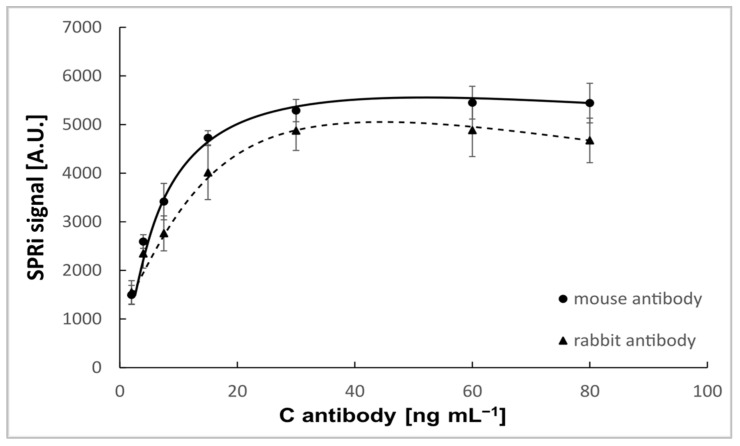
Dependence of SPRI signal (Arbitrary Units) on mouse and rabbit osteopontin antibody concentration. OPN concentration: 10 ng mL^−1^. pH = 7.4.

Mouse monoclonal and rabbit monoclonal antibodies of human osteopontin were chosen as bioreceptors. Antibody solutions were prepared in PBS buffer with concentrations of 2.00–80 ng mL^−1^ (pH = 7.4). After immobilizing the antibody, the biosensor was treated with OPN solution at a constant concentration (10 ng mL^−1^). For immunoassays, it is common to use buffers with a pH around 7.4 (physiological pH) to avoid disrupting the protein’s structure [23]. The measurement procedure is described in Section 2.2. The results are given in Figure 3. As the antibody concentration increases, the SPRi signal also increases until it reache sa constant level. Above 30 ng mL^−1^, antibody binding no longer occurs on the biosensor surface. An antibody concentration of 30 ng mL^−1^ was chosen for additional research.

#### 3.2.2. Optimization of Time Interaction

**Figure 4:**
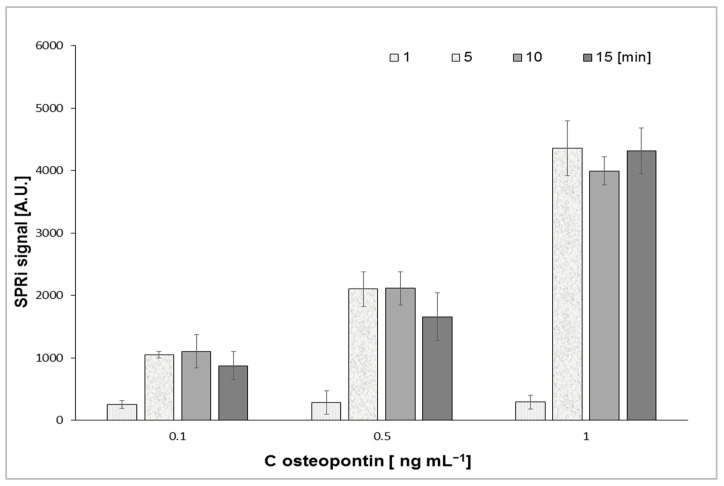
Dependence of the SPRi signal on the interaction time of the antibody and osteopontin at different OPN concentrations.

The optimization of interaction time was evaluated for the efficient binding of the antigen with the antibody. To successfully estimate the optimum interaction time, the biosensor was exposed to a droplet osteopontin solution of 0.1, 0.5, and 1 ng mL^−1^ at 1, 5, 10, and 15 min. Figure 4 shows the dependence of the SPRi signal on the time of interaction of osteopontin with a mouse monoclonal antibody. A similar result was obtained for the second antibody tested. Five minutes was chosen as the optimal interaction time of the analyte with the antibody because the signal stabilized at a constant level after this time.

### 3.3. Estimating the Calibration Range

**Figure 5:**
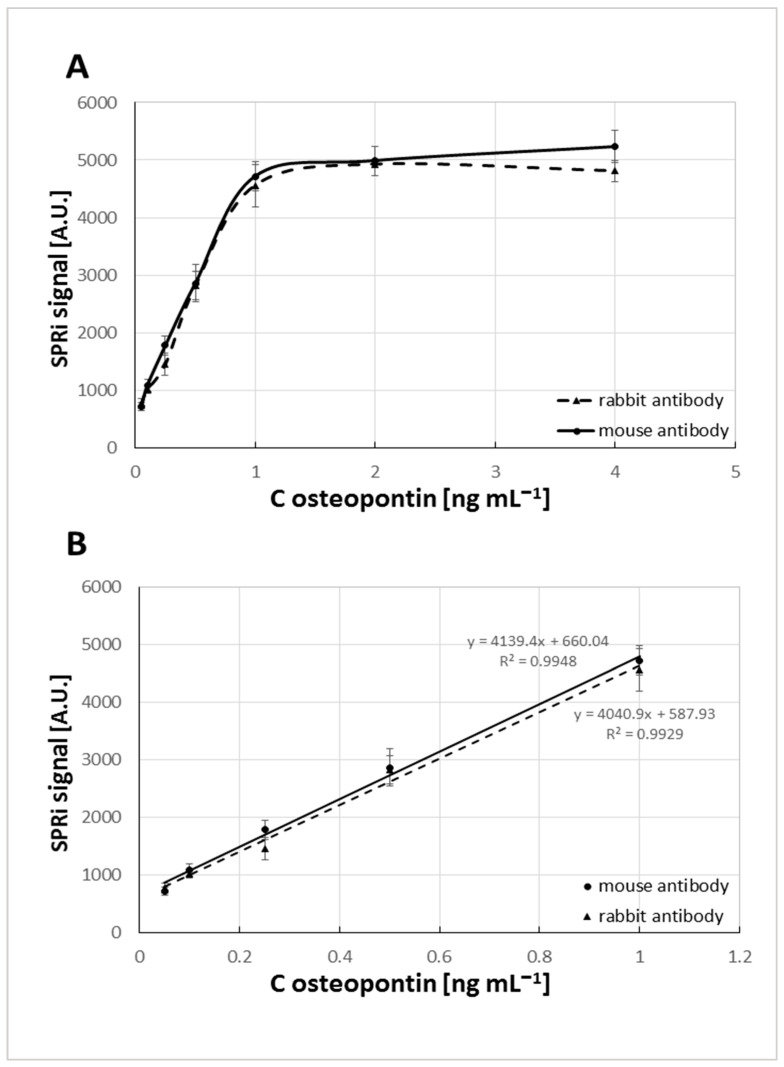
Dependence of SPRi signal (Arbitrary Units) on osteopontin concentration (**A**) in the full range of tested concentrations; (**B**) the calibration curve. Antibody concentration: 30 ng mL^−1^. pH = 7.4.

The dependence of the SPRi signal on the osteopontin concentration was obtained using a series of osteopontin standard solution dilutions in PBS buffer (pH = 7.4) to replicate the typical conditions of human blood serum. The concentration range from 0.05 to 4 ng mL^−1^ was tested. It can be observed that the intensity of the SPRi signal increases with the concentration of the osteopontin within the concentration range studied (Figure 5), reaching a plateau above 1 ng mL^−1^ for both bioreceptors used. Experiments showed that SPRi analysis could achieve a good linear regression in the concentration range of 0.05–1.00 ng mL^−1^ with a regression coefficient of 0.9948 for the mouse antibody and 0.9929 for the rabbit antibody. The LOD and LOQ were determined by calculations using the formulas LOD = 3.3 SD/A and LOQ = 10 SD/A, where SD was the standard deviation measured for the blank control and A was the slope of the calibration curve. The obtained LOD values were LOD = 0.014 ng mL^−1^, LOQ = 0.043 ng mL^−1^ for the biosensor with mouse antibody as the capture element and LOD = 0.018 ng mL^−1^, LOQ = 0.055 ng mL^−1^ with the rabbit antibody as the capture element.

### 3.4. Precision, Accuracy, and Recovery of the SPRi Method for the Determination of Osteopontin

The precision and accuracy of SPRi biosensors sensitive to OPN were determined by the threefold measurement of the concentrations of standard solutions with the following concentrations: 0.05, 0.25, 0.50 ng mL^−1^ and 1 ng mL^−1^. The obtained standard deviations (SDs) and coefficients of variation (CVs) (Table 1) indicate high repeatability and good precision for the measurements performed using both biosensors. However, slightly better parameters were obtained using the mouse antibody as a bioreceptor.

A spike-and-recovery experiment was performed for nine plasma samples for the recovery study. A known concentration of OPN (4 ng mL^−1^) was added to the sample matrix, and whether the found concentration was identical to the real concentration was observed. Measurements were performed for both SPRi biosensors. The recovery was 93–107% for the biosensor with mouse antibody as a bioreceptor and 92–111% for the biosensor with rabbit antibody as a bioreceptor (Table 2). Comparing the concentrations measured by both biosensors, a correlation coefficient 0.97 was obtained.

### 3.5. Interference Studies

The majority of non-specific molecules are found in clinical samples. These naturally occurring biomolecules could potentially interact with the bioreceptor and interfere with the signal in an unwanted way. Since OPN is an element of ECM, fibronectin and collagen were used as potential interfering substances. The influence of albumin, the most abundant protein in plasma, on the quantitative determination of osteopontin was also examined. The experiments were performed at a constant OPN spike and 10 and 100 times excess of the fibronectin and collagen IV and 100 and 1000 times excess of the albumin as interferents. The results are presented in Table 3.

The results show that none of the tested proteins significantly influences the measurement of OPN concentration. The mean recovery is 103 ± 2%, and the mean CV is 4.5%.

### 3.6. Determination of Osteopontin Concentration in Biological Samples

**Figure 6:**
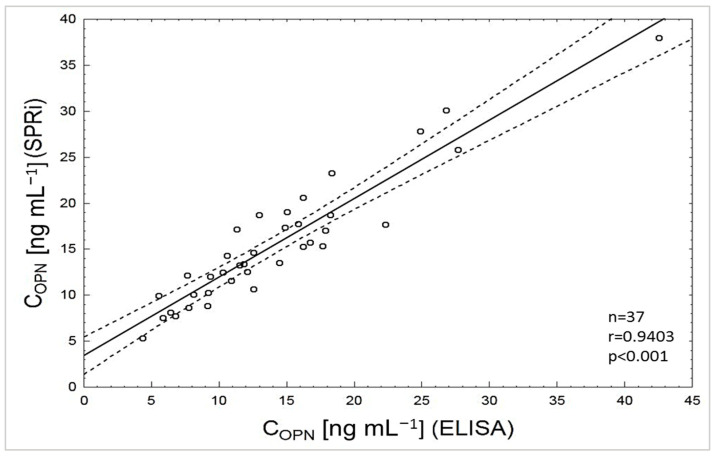
Correlation between plasma OPN levels quantified with the SPRi biosensor and the traditional ELISA assay. Each point represents a single patient. The plot of correlation (—), confidence interval ( - - - ).

Our SPRi method’s final test for OPN detection was its application to the quantification of OPN in patient samples previously analyzed by the standard ELISA reference technique. The biosensor SPRi with mouse antibody as a bioreceptor was applied to test 37 available samples taken from patients with diagnosed benign prostate hyperplasia (BPH) and bladder cancer (BC). The samples were diluted so that the OPN concentrations were within the range of the obtained calibration curve. The values for OPN concentration in plasma measured using the SPRi biosensor and the ELISA technique were compared, and a Spearman correlation coefficient of 0.94 was obtained (Figure 6).

The obtained concentration values, measured by the SPRi biosensor, were 5.4–17.8 ng mL^−1^ (mean value 16.6 ± 4.2 ng mL^−1^) for BC samples and 7.5 ± 38.0 ng mL^−1^ (mean value 10.6 +/− 4.2 ng mL^−1^) for BPH samples. Plasma OPN concentrations in patients with bladder cancer were statistically significantly higher (*p* = 0.033) than in patients with BPH. Similar values for OPN concentrations in plasma were obtained in studies conducted by Lanteri et al. (43.96 ± 9.10 ng mL^−1^) [24], Przepiórka-Kosińska et al. (31.65 ng mL^−1^ on average in psoriatic patients and 11.42 ng mL^−1^ in healthy volunteers) [25], and Chang et al. (range of 9.85–56.95 ng mL^−1^ in peritoneal dialysis patients) [26].

## 4. Conclusions

In this paper, we have described the development of two SPRi biosensor assays to detect osteopontin by employing a mouse or rabbit antibody as a selective biomolecular receptor. The formation of successive biosensor layers was confirmed by plotting SPR curves, and their shifts towards higher angle values confirmed the formation of successive layers on the biosensor’s surface. Under optimized conditions, both biosensors have linear response ranges suitable for determining OPN in biological samples, with LOQ = 0.043 ng mL^−1^ for the biosensor with mouse antibody as the capture element and LOQ = 0.055 ng mL^−1^ with the rabbit antibody as the capture element. The proposed SPRi biosensors allow us to determine the OPN level with good precision and accuracy, as evidenced by the small SD of concentrations determined in samples and CV values not exceeding 8%. The usefulness of the constructed biosensors for determining OPN was tested by measuring the OPN concentration in plasma samples from patients suffering from benign prostate hyperplasia and patients with bladder cancer. The agreement of the obtained results with the determination of OPN concentrations by the standard test ELISA was also checked, and a good correlation was obtained between both methods. A significant result of this study is the development of a method with a simple procedure with non-toxic reagents that can be used to determine OPN in small-volume (2–3 µL) samples. The test time from the moment of bioreceptor preparation for immobilization to the actual measurement is about 1 h. The measurement itself takes about 15 min. It does not require tags, which allows the preservation of the functional properties of the biomolecules to be determined. In conclusion, we developed two methods based on SPRi biosensors to detect osteopontin by employing antibodies as selective receptors that could be used for the quantification of OPN not only in serum but also possibly in other bodily fluids.

## Figures and Tables

**Table 1:** Precision and accuracy of the developed method.

Biosensor with Mouse Antibody					Biosensor with Rabbit Antibody			
C Added [ng mL^−1^]	C Found Mean [ng mL^−1^]	SD	Recovery [%]	CV [%]	C Found Mean [ng mL^−1^]	SD	Recovery [%]	CV [%]
0.050	0.046	0.004	93	7.6	0.049	0.004	99	7.3
0.250	0.248	0.002	99	0.7	0.258	0.010	103	3.9
0.500	0.521	0.008	104	1.4	0.509	0.014	102	2.8
1.000	0.981	0.017	99	1.7	1.016	0.035	102	3.4

**Table 2:** Recovery test for OPN in human plasma using the SPRi biosensors.

Bioreceptor	Nr Samples	OPN Concentration [ng mL^−1^]			
		Plasma Not Spiked with Osteopontin	Expected	Observed	Recovery [%]
Mouse antibody	1	12.0	16.0	15.9	99
	2	12.6	16.6	17.1	103
	3	11.6	15.6	16.7	107
	4	10.3	14.3	13.5	94
	5	13.5	17.5	17.3	99
	6	18.7	22.7	21.1	93
	7	10.1	14.1	13.9	99
	8	15.3	19.3	20.2	105
	9	7.8	11.8	12.2	104
Rabbit antibody	1	12.8	16.8	15.6	93
	2	13.1	17.4	17.9	105
	3	12.8	16.8	15.6	93
	4	11.5	15.5	14.4	93
	5	12.3	16.3	16.4	101
	6	17.9	21.9	20.7	95
	7	9.7	13.7	15.2	111
	8	15.4	19.4	17.9	92
	9	8.6	12.6	12.7	101

**Table 3:** Influence of excess human fibronectin, collagen IV, and albumin on the results of the determination of OPN concentration by the biosensors SPRi.

Potential Interferent	OPN Spike [ng mL^−1^]	Excess Over OPN	Found OPN (Mouse Antibody as Bioreceptor [ng mL^−1^]	Recovery [%]	Found OPN (Rabbit Antibody as Bioreceptor [ng mL^−1^]	Recovery [%]
Fibronectin	0.50	1:10	0.514 ± 0.022	103	0.518 ± 0.011	102
	0.50	1:100	0.501 ± 0.012	100	0.514 ± 0.018	103
Collagen IV	0.50	1:10	0.515 ± 0.023	103	0.519 ± 0.022	104
	0.50	1:100	0.506 ± 0.034	101	0.506 ± 0.026	101
Albumin	0.50	1:100	0.531 ± 0.029	106	0.528 ± 0.017	106
	0.50	1:1000	0.516 ± 0.038	103	0.518 ± 0.022	104

## Data Availability

The original contributions presented in this study are included in the article. Further inquiries can be directed to the corresponding author.
